# Comparative Transcriptomics Reveals the Molecular Mechanism of the Parental Lines of Maize Hybrid An’nong876 in Response to Salt Stress

**DOI:** 10.3390/ijms23095231

**Published:** 2022-05-07

**Authors:** Xingen Zhang, Jing Liu, Yuanxiang Huang, Hongying Wu, Xiaolin Hu, Beijiu Cheng, Qing Ma, Yang Zhao

**Affiliations:** National Engineering Laboratory of Crop Stress Resistance Breeding, School of Life Sciences, Anhui Agricultural University, Hefei 230036, China; 13145518767@163.com (X.Z.); janetann1996@163.com (J.L.); 13696549319@163.com (Y.H.); why38762022@163.com (H.W.); huxiaolin6899@163.com (X.H.); beijiuchengahau@163.com (B.C.)

**Keywords:** maize, An’nong876, salt stress, RNA-seq

## Abstract

Maize (*Zea* *mays* L.) is an essential food crop worldwide, but it is highly susceptible to salt stress, especially at the seedling stage. In this study, we conducted physiological and comparative transcriptome analyses of seedlings of maize inbred lines An’nong876 paternal (cmh15) and An’nong876 maternal (CM37) under salt stress. The cmh15 seedlings were more salt-tolerant and had higher relative water content, lower electrolyte leakage, and lower malondialdehyde levels in the leaves than CM37. We identified 2559 upregulated and 1770 downregulated genes between salt-treated CM37 and the controls, and 2757 upregulated and 2634 downregulated genes between salt-treated cmh15 and the controls by RNA sequencing analysis. Gene ontology functional enrichment analysis of the differentially expressed genes showed that photosynthesis-related and oxidation-reduction processes were deeply involved in the responses of cmh15 and CM37 to salt stress. We also found differences in the hormone signaling pathway transduction and regulation patterns of transcription factors encoded by the differentially expressed genes in both cmh15 and CM37 under salt stress. Together, our findings provide insights into the molecular networks that mediate salt stress tolerance of maize at the seedling stage.

## 1. Introduction

The growing global population has increased the demand for food. Maize (*Zea mays* L.) is an important food and industrial crop worldwide, but its susceptibility to salt stress has limited its yield [1,2]. The high concentration of Na^+^ in saline-alkali soil can lead to hypertonic stress, as well as osmotic and ionic toxicity effects [3,4,5]. For survival and reproductive success, plants must develop multiple strategies to cope with salt stress [3,4,6]. Many studies on how plants respond to salt stress have been conducted and many advances have been achieved over the years [5,7]. 

Plants use various mechanisms to cope with salt stress, including the salt overly sensitive (SOS) signaling pathway, balance of Na^+^/K^+^ by high-affinity K^+^ transport proteins, reactive oxygen species (ROS) scavenging and plant hormone pathways [3,8,9]. Pei and coworkers [10] were the first to show how Na^+^ was perceived in plants by studying *Arabidopsis* under salt stress. In the *Arabidopsis* mutant monocation-induced Ca^2+^ increases 1 (moca1), they found that salt-induced depolarization of cell surface potential, Ca^2+^ peak and wave, and Na^+^/H^+^ antiporter activation occurred [10]. In the SOS pathway, the calcium-binding EF hand family protein SOS3 interacts with the protein kinase SOS2 and activates it [3,11]. The activated SOS2 can phosphorylate and activate the Na^+^/H^+^ inversion transporter SOS1 on the plasma membrane, which removes Na^+^ from cells and loads Na^+^ into the xylem from where it is transported to the leaves [11,12]. The cation transporter HTK1 is another important Na^+^ transporter [13]. In plant leaves, HTK1 transports Na^+^ into the phloem for recycling back to the roots, and in the roots, HTK1 removes Na^+^ from xylem [14,15]. Under salt stress, plants produce large amounts of ROS, which have strong oxidizing ability and cause irreversible loss of cell membranes [16,17]. Superoxide dismutase (SOD) is the first line of defense to protect plant from ROS damage [18]. Alongside SOD, ascorbate peroxidase and catalase can remove the oxides and help protect the cells [18,19].

Plant hormones not only regulate plant growth and development, but they also mediate a variety of stresses [20,21]. Abscisic acid (ABA) is central to plant development and response to external stress and is involved in the release of Ca^2+^ and the regulation of stomatal closure in response to salt stress [22,23]. Plants also accumulate ethylene in response to salt stress, indicating that ethylene may have an essential role in the response [20]. Spraying exogenous jasmonic acid (JA) can reduce salt toxicity and blocking JA signal transmission was shown to reduce the tolerance of plants to salt stress [24,25,26]. Furthermore, plants can limit their growth and development by reducing gibberellin levels to help them cope with salt stress [27,28]. The levels of other growth hormones, such as cytokinin (CK), auxin/indole-3-acetic acid (AUX/IAA) and brassinosteroids, can also be reduced in response to salt stress [20,29,30,31]. 

Transcriptome sequencing technologies have been used to analyze gene expression and identify candidate genes related to specific traits [2,32,33]. For example, by analyzing the transcriptome of maize under drought conditions, Ka-kumanu et al. [34] found a large number of drought-induced genes in ovary and basal leaf meristem. Maize hybrid An’nong876 suitable for planting in the Huang-Huai-Hai region of China is an excellent new maize variety that has shown strong environment adaptability. Therefore, analyzing the salt response mechanism of paternal and maternal lines of An’nong876 may enrich the understanding of the regulatory mechanism of the maize response to salt stress. In this study, we compared the expression profiles of two maize inbred lines, An’nong876 paternal line (cmh15) and An’nong876 maternal line (CM37), under control and high salt conditions by RNA sequencing (RNA-seq) and identified differentially expressed salt-stress responsive genes. Our results provide new perspectives for understanding the salt stress response mechanism in maize.

## 2. Results

### 2.1. Characteristics of Maize Inbred Lines CM37 and cmh15 

**Figure 1:**
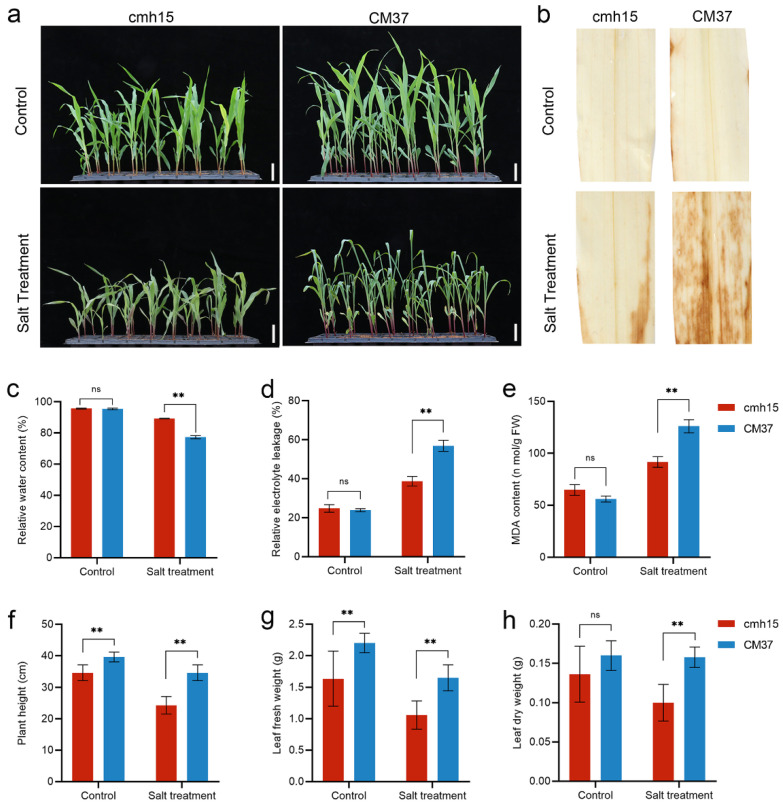
Phenotypic and physiological responses of cmh15 and CM37 seedlings under control and salt stress conditions. (**a**) Phenotypic response of cmh15 and CM37 seedlings. Scale bar = 5 cm. (**b**) DAB staining of CM37 and cmh15 leaves. (**c**–**e**) Relative water content, relative electrolyte leakage, and malondialdehyde content. (**f**–**h**) Plant height, leaf fresh weight, and leaf dry weight. Each bar represents at least three means ± SE. ** *p* ≤ 0.01; ns, not significant (calculated by *t*-test).

CM37 and cmh15 seedlings were treated with 300 mM of NaCl and their phenotypic response to salt stress was evaluated when the third leaf of the seedlings was fully expanded. Under the controlled conditions, the CM37 and cmh15 seedings grew well, but the average height of the CM37 plants was higher than that of the cmh15 plants (Figure 1a,f). There were no differences in the relative water content (RWC), relative electrolyte leakage (REL), and malondialdehyde (MDA) content physiological indicators between the CM37 and cmh15 plants (Figure 1c–e). Under the salt stress conditions, the cmh15 plants maintained higher RWC but have lower REL and MDA contents than the CM37 plants (Figure 1c–e). Diaminobenzidine (DAB) staining showed that the CM37 leaves were more deeply stained than the cmh15 leaves (Figure 1b). The plant height, fresh weight, and dry weight were decreased in the salt-treated CM37 and cmh15 plants compared with controls (Figure 1f–h).

### 2.2. RNA-seq Analysis and Identification of DEGs

**Figure 2:**
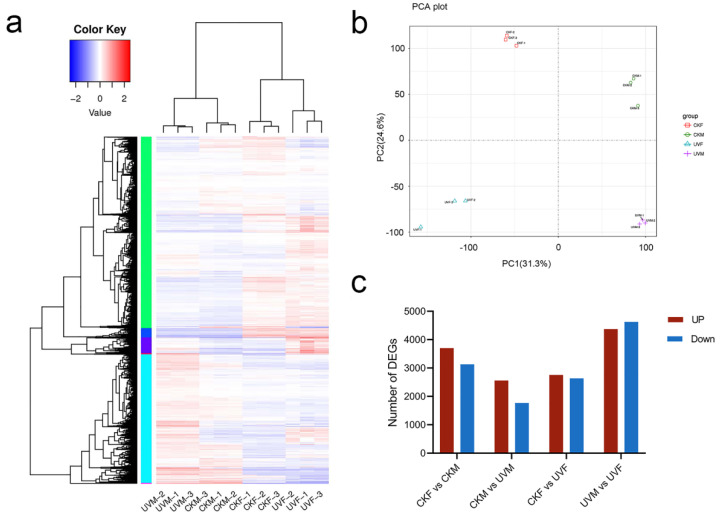
Differentially expressed genes (DEGs) between cmh15 and CM37 under control and salt stress conditions. (**a**) Heatmap showing the clustering of salt-stress responsive genes. (**b**) Principal component analysis of cmh15 and CM37 under control and salt treatment conditions. (**c**) DEGs detected in the 4 comparisons among the control and salt stress treatment groups.

To identify genes responsive to salt stress, we performed RNA-seq and analyzed the data to obtain the whole genomic expression of CM37 and cmh15. After removing low quality sequences and the adapter sequence, we obtained an average of 44.7 million reads with a length of 150 bp from each sample (Table S1). There was a total of 12 samples, CKM (CM37 control), CKF (cmh15 control), UVM (salt-treated CM37) and UVF (salt-treated cmh15), with 3 replicates for each group. Among the clean reads, 75.80–78.94% were unique and were mapped to the maize B73 reference genome AGPv4 (Table S1). The expression level of each gene was normalized by the fragment per kilobases per million reads (FPKM) method. The overall gene expression levels were higher after salt stress compared with their levels under controlled conditions (Figure 2a). Principal component analysis of cmh15 and CM37 was performed to examine the relationship and variation among them. The first and second principal components accounted for 31.3% and 24.6% of the variance, respectively. The three biological replicates of each group clustered together, which confirmed that the expression profiles of the replicates were highly correlated (Figure 2b). Genes were considered to be significantly differentially expressed genes (DEGs) when the fold change was ≥2 and the adjusted *p* value was ≤0.05. In the CKM vs. CKF comparison (CKM as the control), we identified 3704 upregulated and 3132 downregulated genes, and, in the UVM vs. UVF comparison (UVM as the control), we identified 4372 upregulated and 4628 downregulated genes. In the CKM vs. UVM (CKM as control) and CKF vs. UVF comparisons (CKF as control), we identified 2559 and 2757 upregulated genes and 1770 and 2634 downregulated genes, respectively (Figure 2c). 

**Figure 3:**
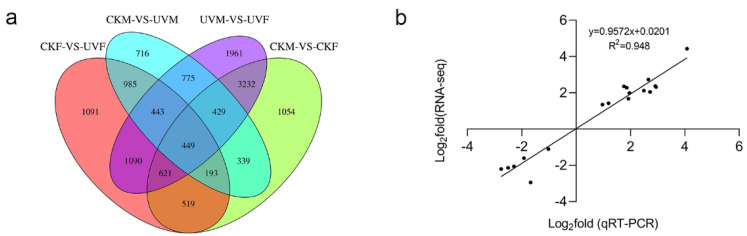
Venn diagram of differentially expressed genes in the four comparisons and validation of the RNA-seq results. (**a**) Numbers of genotype-specific and common DEGs. (**b**) Correlation analysis between the RNA-seq and qRT-PCR results.

We compared the DEGs detected in the four comparisons to investigate their roles in the salt stress response. We found that the overlapping DEGs were of two types, genotype-specific responsive genes and common salt-stress responsive genes (i.e., common between genotypes). In the 4 comparisons, we identified 716 (CKM vs. UVM) and 1091 (CKF vs. UVF) genotype-specific DEGs (Figure 3a). Because the genetic backgrounds of cmh15 and CM37 differ considerably, we focused on the CKM vs. UVM and CKF vs. UVF comparisons in all subsequent analyses. 

To verify the reliability of the RNA-seq data, we selected nine DEGs for qRT-PCR analysis (Figure S1). The qRT-PCR results showed high correlation with the RNA-seq results (R^2^ = 0.948), which confirmed the expression results were reliable (Figure 3b).

### 2.3. Gene Ontology and Kyoto Encyclopedia of Genes and Genomes Pathway Functional Enrichment Analysis of DEGs

**Figure 4:**
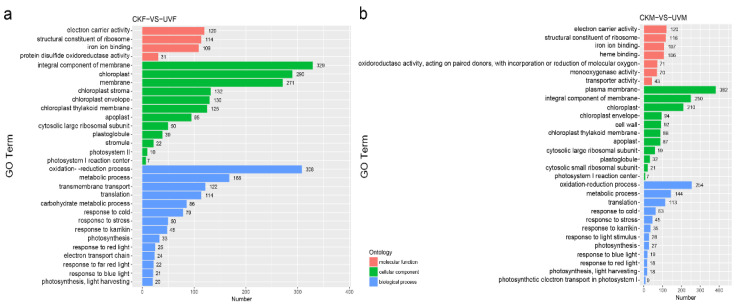
Gene ontology (GO) enrichment analysis of the DEGs detected in the CKF vs. UVF (**a**) and CKM vs. UVM (**b**) comparisons. The topmost enriched GO terms under the three main GO categories are shown.

The roles of the salt-stress responsive DEGs in the CKM vs. UVM and CKF vs. UVF comparisons were predicted by gene ontology (GO) enrichment analysis. Terms related to oxidation-reduction and metabolism were enriched in cmh15 and CM37 under salt stress; for example, oxidation-reduction process (GO:0055114) was highly enriched (Figure 4a,b). Terms associated with photosynthesis were also highly enriched in cmh15 and CM37 under salt stress, including photosynthesis (GO:0009765), light harvesting (GO:0009765), response to red light (GO:0010114), response to blue light (GO:0009637), and photosystem (GO:0009538) (Figure 4a,b). Terms related to protein translation were also highly enriched, including translation (GO:0006412) and structural constituent of ribosome (GO:0003735). Many enriched GO terms were associated with stress response, including response to stress (GO:0006950) and response to cold (GO:0009409) (Figure 4a,b). The GO terms carbohy-drate metabolic (GO:0005975), transmembrane transport (GO:0090662), response to far red light (GO:0010218) and electron transport chain (GO:0022900) were more highly enriched in the CKF vs. UVF comparison than they were in the CKM vs. UVM comparison.

**Figure 5:**
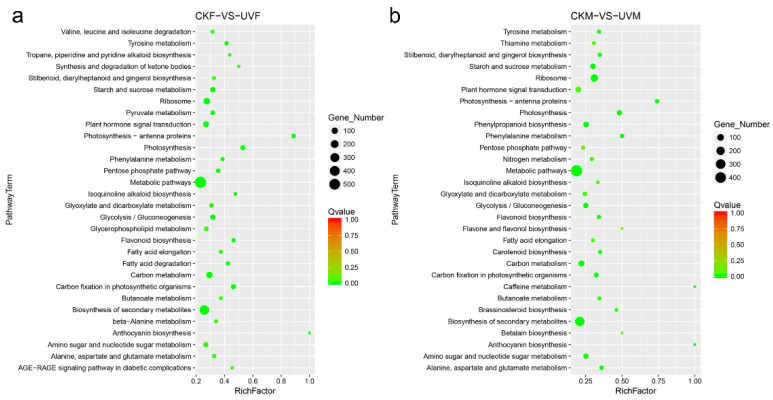
KEGG pathway enrichment analysis of the DEGs detected in the CKF vs. UVF (**a**) and the CKM vs. UVM (**b**) comparisons. The significance of the enriched pathways is based on *q* values < 0.05. Rich Factor is the ratio of DEGs to the total number of genes in each pathway.

In the Kyoto Encyclopedia of Genes and Genomes (KEGG) pathway *enrichment analysis*, degradation of ketone bodies (ko00072), pyruvate metabolism (ko00620), glycer-ophospholipid metabolism (ko00564), fatty acid degradation (ko00071), anthocyanin biosynthesis (ko00942), beta-alanine metabolism (ko00410), and AGE-RAGE signaling pathway (ko04933) were the top most enriched pathways in cmh15 under salt stress, whereas thiamine metabolism (ko00730), phenylalanine biosynthesis (ko00400), glycolysis/gluconeogenesis (ko00010), carotenoid biosynthesis (ko00906), and carotenoid biosynthesis (ko00906) were the topmost enriched pathways in CM37 under salt stress (Figure 5a,b). Starch and sucrose metabolism (ko00500), ribosome (ko03008), plant hormone signal transduction (ko04075), photosynthesis (ko00195), photosynthesis-antenna proteins photosynthesis (ko00196), metabolism pathways (ko01100), pentose phosphate pathway (ko00030), phenylalanine metabolism (ko00360), flavonoid biosynthesis (ko00941), isoquinoline alkaloid biosynthesis (ko00950), glyoxylate and dicarboxylate metabolism (ko00630), fatty acid elongation (ko00062), carbon metabolism (ko01200), biosynthesis of secondary metabolism (ko00999), anthocyanin biosynthesis (ko00942), and amino sugar and nucleotide sugar metabolism (ko00520) were enriched in cmh15 and CM37 (Figure 5a,b).

### 2.4. Analysis of DEGs Encoding Transcription Factors

**Figure 6:**
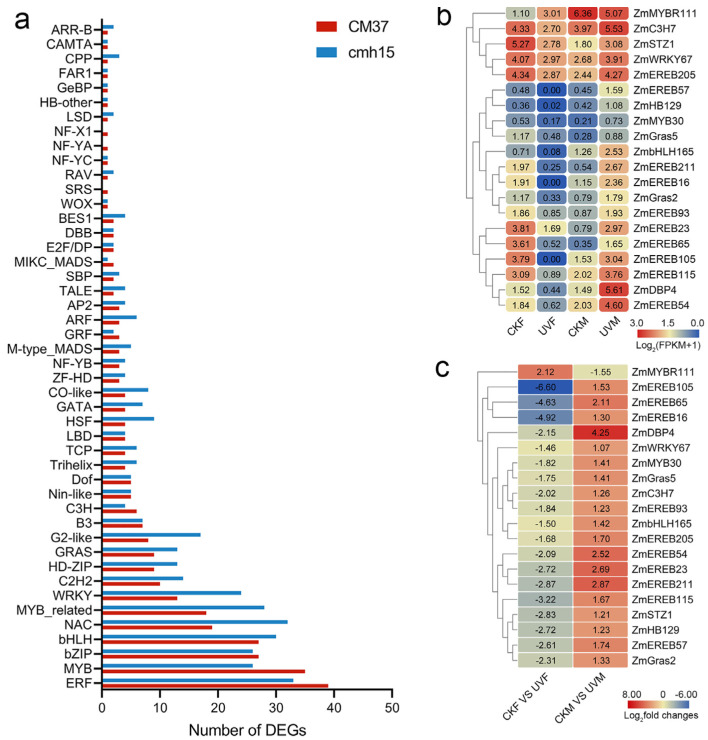
Transcription factors (TFs) encoded by DEGs detected in the CKM vs. UVM and CKF vs. UVF comparisons. (**a**) Classification and statistics of the TF families. (**b**,**c**) Heatmaps of the FPKM+1 values (**b**) and fold changes (**c**) of the 20 TFs that showed opposite regulatory trends in the two comparisons.

A total of 304 transcription factors (TFs) that belonged to 46 TF families were encoded by DEGs detected in the CKF vs. UVF comparison, and 375 TFs that belonged to 44 TF families were encoded by DEGs detected in the CKM vs. UVM comparison (Figure 6a). The families with the highest numbers of TFs were ERF, MYB, bZIP, bHLH, NAC, and WRKY (Figure 6a). We found that 132 of the TFs were common in both comparisons; 112 of them showed consistent regulatory trends and the remaining 20 TFs showed opposite regulatory trends (Figure 6b,c). Among the common TFs, *ZmEREB211* and *ZmMYB30* have been reported as stress-related candidate gene [35,36], and *ZmDBP4* and *ZmWRKY114* were confirmed to be involved in the stress response [37,38]. The number of TFs exhibited significant differences between CKF vs. UVF and CKM vs. UVM comparisons. For example, as shown in Figure 6a, the NAC transcription factor family has 19 members in CKM vs. UVM comparison and 32 members in CKF vs. UVF comparison.

### 2.5. Analysis of DEGs Associated with Plant Hormone

**Figure 7:**
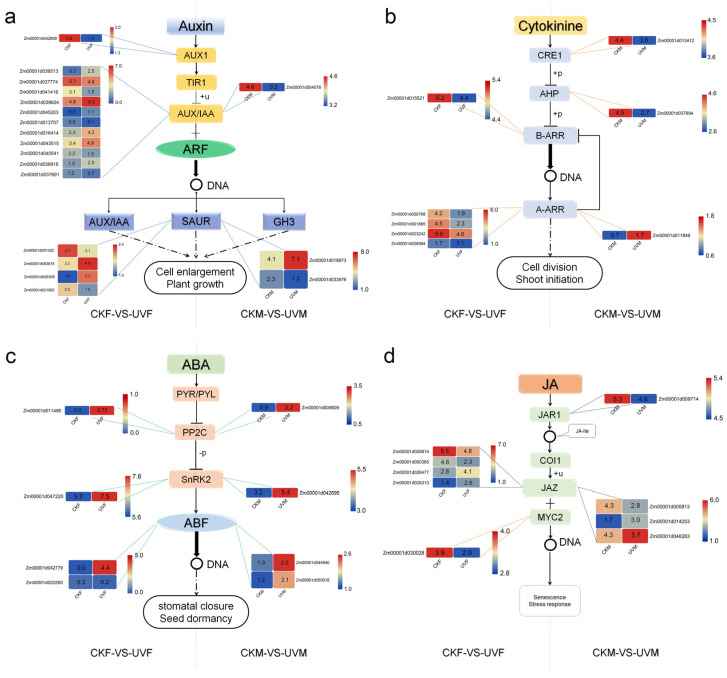
Hormone signal transduction pathways of genotype-specific DEGs in the CKM vs. UVM or CKF vs. UVF comparisons. (**a**) Auxin signal transduction pathway. (**b**) Cytokinin signaling pathway. (**c**) ABA signaling pathway. (**d**) JA signaling pathway. The colored bars indicate the numbers of DEGs for CM37 and cmh15 in the control and salt-treated groups (Log_2_ (FPKM + 1).

To identify crucial genes involved in important pathways, differentially expressed genes of the CKM vs. CKF comparison under controlled conditions were removed from CKF vs. UVF comparison or CKM vs. UVM comparison. In the KEGG analysis, some of the DEGs were enriched in IAA, CK, ABA, and JA signaling pathways. A total of 3 DEGs in the CKM vs. UVM comparison, *Zm00001d018973 (IAA24)*, *Zm00001d033976 (IAA4)*, and *Zm00001d004578 (SAUR50)*, and 16 DEGs in the CKF vs. UVF comparison were associated with the AUX/IAA signaling pathway. Among them, *Zm00001d042809 (ATL1)*, located upstream of the signal transduction pathway, was downregulated, which suggests that transmission of the auxin signal may be inhibited at the upstream. Among the other 15 DEGs, there were 6 upregulated and 5 downregulated DEGs that encode AUX/IAA type proteins, and 2 upregulated and 2 downregulated DEGs that encode SAUR type proteins (Figure 7a). For DEGs associated with the CK signal pathway, *Zm00001d013412 (CRE1)* and *Zm00001d037694 (AHP4)* were downregulated and *Zm00001d011849 (A-AAR4)* was upregulated in the CKM vs. UVM comparison, and one DEG that encoded B-ARR and four DEGs that encoded A-ARR were down regulated in the CKF vs. UVF comparison (Figure 7b). Together, these results show that, under salt stress, the auxin signal transduction pathway of cmh15 was more affected than that of CM37, and cmh15 was greatly affected in the downstream of the cytokinin signal transduction pathway, whereas CM37 was affected to a smaller extent in the upstream.

Similarly, there were four DEGs associated with the ABA signaling pathway in the CKM vs. UVM comparison (namely *Zm00001d005609* and *Zm00001d042695*, which encoded PP2C type protein and SnRK2.4 type protein, respectively, and *Zm00001d044940 (bZIP100)* and *Zm00001d050018 (bZIP68))* all four genes were upregulated (Figure 7c). Four DEGs related to ABA signal transduction were also detected in the CKF vs. UVF comparison; *Zm00001d011495 (PP2C15)* and *Zm00001d047220* (SnRK2.1) were upregulated, and *Zm00001d042779*, which encodes an ABF type protein, was highly upregulated and *Zm00001d022550 (bZIP92)* was slightly downregulated (Figure 7c). Among the four DEGs associated with the JA signaling pathway that were detected in the CKM vs. UVM comparison, *Zm00001d009714*, which is upstream of signal transduction, was downregulated. The other three DEGs encode JAZ type proteins, namely *Zm00001d005813 (TIFY15)*, which was downregulated, and *Zm00001d014253 (TIFY20)* and *Zm00001d048263 (TIFY28)*, which were upregulated. Five DEGs associated with the JA signaling pathway were detected in the CKF vs. UVF comparison. Four of them, *Zm00001d026477 (ZIM34)*, *Zm00001d028313*, *Zm00001d020614 (ZIM28)*, and *Zm00001d050365 (TIFY17)*, encode *JAZ* type proteins, the former two were upregulated and the latter two were downregulated (Figure 7d). The other DEG *Zm00001d030028* which encodes a MYC2 type protein, was downregulated. In the JA signaling pathway, *JAZ* and *MYC2* interact with other hormone signal components, which may relate to plants’ tolerance to salt stress [39]. 

### 2.6. Analysis of DEGs Associated with Photosynthesis

**Figure 8:**
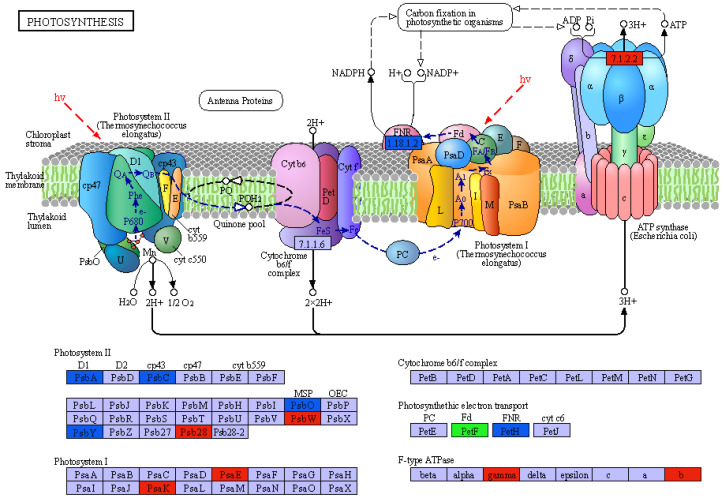
KEGG map of the photosynthesis pathway. Blue and red boxes indicate the specific steps that the DEGs in cmh15 and CM37, respectively, were involved in. The green box indicates that DEGs in both CM37 and cmh15 were involved in this step.

In the GO analysis, some of the DEGs were annotated with photosynthesis (GO:0009765), light harvesting (GO:0009765), and in the KEGG analysis, DEGs were enriched in photosynthesis (ko00195) and photosynthesis-antenna proteins (ko00196). In the CKF vs. UVF and CKM vs. UVM comparisons, 34 and 33 DEGs were associated with photosynthesis, respectively; 26 of the DEGs were common and all of them were downregulated (Table S2). The DEGs affect mainly photosynthetic system I (PS I) and photosynthetic system II (PS II). PS I and PS II absorb photons of different bands for energy transfer, and are important in the whole photosynthesis process [40]. The common DEGs were downregulated, but the downregulation in the CKF vs. UVF comparison was more than it was in the CKM vs. UVM comparison (Table S2). The specific DEGs in cmh15 were associated with PSI and PSII (Figure 8, Table S3), whereas the specific DEGs in CM37 were associated mainly with PS II (Figure 8, Table S4). 

## 3. Discussion

Salt stress is one of the main abiotic stresses limiting maize yield [41,42]. Different plants and even different inbred line of the same plant have been shown to adapt to salt stress in different ways [42,43]. Therefore, analyzing the salt response in different germplasm of the same plant can increase the understanding of how plants respond to high salt conditions. We used the CM37 and cmh15 maize inbred lines, which showed different resistance to salt stress (Figure 1), and investigated the common and specific molecular mechanisms associated with their response to salt stress by transcriptome analysis. 

We identified 5391 and 4329 DEGs before and after salt treatment in cmh15 and CM37, respectively. The GO enrichment analyses of the DEGs showed that photosynthesis-related reaction processes were severely affected in cmh15 and CM37 in response to salt stress, which is consistent with the findings in rice and wheat that showed that salt stress significantly impacted photosynthesis in these plants [44,45]. We found that most of the DEGs related to photosynthesis were downregulated, and that the common DEGs were more downregulated in the CKF vs. UVF comparison than they were in the CKM vs. UVM comparison (Table S2). 

Plant hormones have important roles in plant growth and development and in the response to abiotic stresses [20,21]. After exposure to salt stress, endogenous ABA levels in plants have been shown to increase rapidly, thereby enhancing the regulation of stomatal opening and closing and increasing the synthesis of osmoregulatory substances [23,46,47]. We found that most of the DEGs associated with ABA were upregulated in CM37 and cmh15 after salt treatment, suggesting that the ABA signaling pathway has an important role in the salt stress response. *JAZ* and *MYC2* in the JA signaling pathway, are essential for the response of plants to salt stress [39], and overexpression of *JAZ* will improve the transgenic plants’ tolerance to salt stress [48]. We found that the two DEGs that encode JAZ type proteins were upregulated in the CKM vs. UVM and CKF vs. UVF comparisons, which may help the plants cope with salt stress. *Zm00001d030028*, which encodes the MYC-type protein MYC7, was downregulated in the CKF vs. UVF comparison, and no MYC-type DEGs were found in the CKM vs. UVM comparison. In Arabidopsis under salt stress, *AtMYC2* was shown to play an important role in inhibiting the JA signal activator on the elongation of primary root cells [49]. We found that the numbers of auxin- and CK-related DEGs varied greatly in cmh15 and CM37. Our data suggest that the auxin and CK signaling pathways may be more affected in cmh15 than they were in CM37 (Figure 7a,b). The reduction in growth hormone accumulation and the inhibition of growth hormone receptor expression to maintain a low growth hormone signaling response state, have been shown to be important for plants to cope with salt stress [50,51,52].

TFs are known to play important roles in plants’ response to salt stress. We found that there more DEGs that encode WRKY, NAC, MYB-related, ERF, G2-Like, GARS, and HD-ZIP TF families in cmh15 than there were in CM37 under salt stress (Figure 7). WRKY TFs had been confirmed to play an important role in the response to salt stress. In maize, *ZmWRKY17* and *ZmWRKY114* are negative regulators that reduce the tolerance of trans-genic crops to salt stress [38,53]. In our study, *ZmWRKY114* was upregulated in CM37 and downregulated in cmh15, which is consistent with its known function. Plant CCCH zinc finger proteins play important roles in plants’ response to abiotic stress. The *AT2G40140* (*AtSZF2*) *Arabidopsis* mutant showed increased expression of salt-stress responsive genes under high salt stress, indicating that *AtSZF2* negatively regulates the tolerance of *Arabidopsis* to salt stress [54]. *Zm00001d010956*, a homolog of *AtSZF2* in maize, was upregulated in CM37 and downregulated in cmh15.

The genotype-specific DEGs in the CKM vs. UVM and CMF vs. UVF comparisons were annotated with GO terms and KEGG pathways (Figure S2). We found that the DEGs involved in mitogen-activated protein kinase (MAPK) cascade pathway were enriched in CKF vs. UVF comparison. In *Arabidopsis* and rice, the MAPK signaling pathway has an important role in the plant response to salt stress [55,56]. In addition, the DEGs involved in fatty-acidrelated pathways were also significantly enriched in CKF vs. UVF comparison. In rice, increasing the fatty acid content could enhance the activity of Na^+^/H^+^ transport proteins and thus enhances the tolerance of rice to salt stress [57]. We concluded that the differences of the enriched pathways may be related to the salt-response of the two inbred lines.

**Figure 9:**
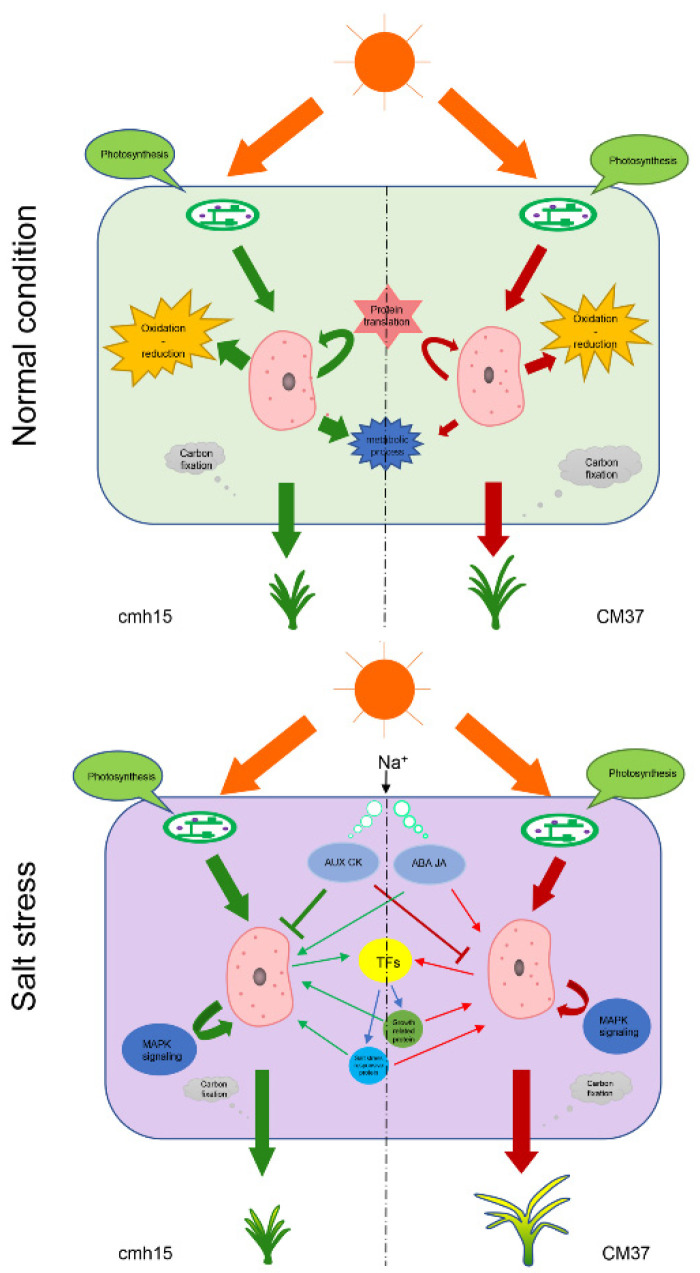
Schematic model illustrating the main processes for obtaining salt tolerance in seedlings of the maize inbred lines CM37 and cmh15. The model is based on the main salt stress responsive DEGs identified in this study.

On the basis of our findings and those reported in the previous studies, we constructed a model to illustrate the differences between cmh15 and CM37 that may be closely related to their growth and response to salt stress. This model may provide a basis for future studies into the salt stress mechanisms and for breeding salt-tolerance germplasm in maize (Figure 9).

## 4. Materials and Methods

### 4.1. Plant Growth and Salt Treatment 

The seeds of the CM37 and cmh15 inbred lines were provided by Professor Qing Ma. The seeds were selected and planted in a greenhouse with a 28 °C/23 °C 16-h light/8-h dark cycle. When the seedlings reached the three-leaf stage, they were irrigated with 300 mM of NaCl every 2 days for 1 week. The seedlings in the control group were irrigated normally. After one week of treatment, the third leaf of each sample was collected in liquid nitrogen and stored at −80 °C for RNA-seq.

### 4.2. Determination of RWC, REL MDA and Protocol of DAB Staining

The RWC and REL of the control and salt-treated seedlings were measured as described previously [58]. MDA content was measured using the MDA assay kit according to the manufacturer’s instructions (Jiancheng Bioengineering Institute, Nanjing, China). Hydrogen peroxide accumulation was measured by DAB staining (Jiancheng Bioengineering Institute) according to the manufacturer’s protocol. Plant height, fresh weight, and dry weight of the control and salt-treated CM37 and cmh15 plants were measured.

### 4.3. Extraction of RNA and Construction of cDNA Library

Total RNA of each sample was extracted using a TRIzol Reagent Mini Kit (Qiagen ChinaCo., Ltd, Shanghai, China). The extracted RNA was quantified using an Agilent 2100 Bioanalyzer (Agilent Technologies, Palo Alto, CA, USA) and qualified by NanoDrop (Invitrogen, San Diego, CA, USA). Total RNA (1 μg) with RNA integrity number values > 7 was used for cDNA library construction. Poly(A) mRNA was isolated using the NEBNext^®^ Poly(A) mRNA Magnetic Isolation Module (NEB, Lpswich, MA, USA). The cDNA libraries were prepared using a NEBNext^®^ Ultra™ RNA Library Prep Kit for Illumina^®^ according to the manufacturer’s instructions (New England Biolabs Inc.). Multiplexed libraries with different indices were sequenced on an Illumina HiSeq platform (Illumina, San Diego, CA, USA) according to the manufacturer’s instructions.

### 4.4. Sequence Assembly and Data Analysis

The raw reads were filtered to obtain high-quality clean reads by removing adapter sequences, sequences that contained poly(N), and short sequences < 75 bp. HISAT2 (v2.0.1) was used to aligning the clean reads to the maize B73 reference genome (RefGen_v4) [59]. The expression levels of the genes in the paired-end clean data were estimated using HTSeq (v0.6.1) [60]. The gene expression level was normalized by the FPKM method [61]. The DESeq2 Bioconductor package was used for differential expression analysis [62]. Genes with fold change ≥ 2 and adjusted *p* value ≤ 0.05 were considered to be differentially expressed.

### 4.5. Validation of RNA-seq Data by qRT-PCR

We selected nine DEGs for validation by qRT-PCR. Single-stranded cDNA was obtained using Evo MMLV RT Premix for qRT-PCR (Accurate Biotechnology Co., Ltd., Changsha, China) according to manufacturer’s protocol. Gene special primers were designed using Pri-mer3Plus (http://www.primer3plus.com/, accessed on 10 January 2021). FastStart Essential DNA Green Master (Roche, Basel, Switzerland) was used for the PCRs. The reaction system and procedure are described in a previous paper [63]. The maize *GAPDH* gene (accession number: NM_001111943.1) was used as the internal control for normalization, and three technical replicates of each cDNA sample were analyzed. The primer sequences for qRT-PCR are listed in Table S5. The 2^−ΔΔCT^ method was used to calculate the relative expression level of each gene [64].

### 4.6. GO and KEGG Enrichment Analysis

The GO enrichment analysis was performed using GOSeq (v1.34.1). The KEGG enrichment analysis was performed using KEGG web service (http://www.kegg.jp/, accessed on 20 November 2020). GO terms and KEGG pathways with adjusted *p* values ≤ 0.05 were considered as significantly enriched.

### 4.7. Statistical Analysis

The SPSS Statistics 19.0 was used to analyze the data of each group.

## 5. Conclusions

We analyzed the RNA-seq to provide a global view of differences in the transcriptomes of maize inbred lines cmh15 and CM37 under normal and salt-stress conditions. A total of 5391 and 4329 DEGs were identified in the 2 inbred lines with and without salt stress, respectively. Analysis of the DEGs showed that salt stress severely affected photosynthesis and oxidation-reduction processes in the plants. We found that the regulatory role of TFs and phytohormone signaling pathways were very important in the response of cmh15 and CM37 to salt stress. Our results show that there were similarities and differences in the responses of cmh15 and CM37 to salt stress, and the findings will provide new perspectives on the salt mechanisms of maize.

## Data Availability

The raw data supporting the conclusions in this article are available in the GenBank GEO database under accession number PRJNA794297 (https://www.ncbi.nlm.nih.gov/sra/PRJNA794297, accessed on 26 March 2022).

## Supplementary Materials

The following supporting information can be downloaded at: https://www.mdpi.com/article/10.3390/ijms23095231/s1.

## Abbreviations

ABA	abscisic acid
ABF	ABA-responsive element binding factor
AUX	auxin
CK	cytokinin
CKF	control group of cmh15
CKM	control group of CM37
DAB	diaminobenzidine
DEG	differential expressed gene
GO	gene Ontology
HKT1	high affinity potassium transporter1
JA	jasmonic acid
KEGG	Kyoto Encyclopedia of Genes and Genomes
MAPK	mitogen-activated protein kinase
MDA	malondialdehyde
PCA	principal component analysis
POD	peroxidase
qRT-PCR	quantitative real-time polymerase chain reaction
REL	relative electrolyte leakage
RNA-seq	RNA sequencing
ROS	reactive oxygen species
RWC	relative water content
SA	salicylic acid
SOD	superoxide dismutase
SOS	salt overly sensitive;
UVF	salt treatment group of cmh15
UVM	salt treatment of CM37

## References

[1] Zhang M., Cao Y.B., Wang Z.P., Wang Z.Q., Shi J.P., Liang X.Y., Song W.B., Chen Q.J., Lai J.S., Jiang C.F. (2018). A retrotransposon in an HKT1 family sodium transporter causes variation of leaf Na^+^ exclusion and salt tolerance in maize. New Phytol..

[2] Luo X., Wang B.C., Gao S., Zhang F., Terzaghi W., Dai M.Q. (2019). Genome-wide association study dissects the genetic bases of salt tolerance in maize seedlings. J. Integr. Plant Biol..

[3] Zhu J.K. (2016). Abiotic Stress Signaling and Responses in Plants. Cell.

[4] Endler A., Kesten C., Schneider R., Zhang Y., Ivakov A., Froehlich A., Funke N., Persson S. (2015). A Mechanism for Sustained Cellulose Synthesis during Salt Stress. Cell.

[5] Yang Y.Q., Guo Y. (2018). Unraveling salt stress signaling in plants. J. Integr. Plant Biol..

[6] Yang Y.Q., Guo Y. (2018). Elucidating the molecular mechanisms mediating plant salt-stress responses. New Phytol..

[7] Munns R., Tester M. (2008). Mechanisms of salinity tolerance. Annu. Rev. Plant Biol..

[8] Deinlein U., Stephan A.B., Horie T., Luo W., Xu G.H., Schroeder J.I. (2014). Plant salt-tolerance mechanisms. Trends Plant Sci..

[9] Kudla J., Becker D., Grill E., Hedrich R., Hippler M., Kummer U., Parniske M., Romeis T., Schumacher K. (2018). Advances and current challenges in calcium signaling. New Phytol..

[10] Jiang Z.H., Zhou X.P., Tao M., Yuan F., Liu L.L., Wu F.H., Wu X.M., Xiang Y., Niu Y., Liu F. (2019). Plant cell-surface GIPC sphingolipids sense salt to trigger Ca^2+^ influx. Nature.

[11] Quan R.D., Lin H.X., Mendoza I., Zhang Y.G., Cao W.H., Yang Y.Q., Shang M., Chen S.Y., Pardo J.M., Guo Y. (2007). *SCABP8*/*CBL10*, a putative calcium sensor, interacts with the protein kinase SOS2 to protect *Arabidopsis* shoots from salt stress. Plant Cell.

[12] Shi H.Z., Quintero F.J., Pardo J.M., Zhu J.K. (2002). The putative plasma membrane Na^+^/H^+^ antiporter SOS1 controls long-distance Na^+^ transport in plants. Plant Cell.

[13] Davenport R.J., Munoz-Mayor A., Jha D., Essah P.A., Rus A., Tester M. (2007). The Na^+^ transporter *AtHKT1*;1 controls retrieval of Na^+^ from the xylem in *Arabidopsis*. Plant Cell Environ..

[14] An D., Chen J.G., Gao Y.Q., Li X., Chao Z.F., Chen Z.R., Li Q.Q., Han M.L., Wang Y.L., Wang Y.F. (2017). *AtHKT1* drives adaptation of *Arabidopsis thaliana* to salinity by reducing floral sodium content. PLoS Genet..

[15] Hill C.B., Jha D., Bacic A., Tester M., Roessner U. (2013). Characterization of Ion Contents and Metabolic Responses to Salt Stress of Different *Arabidopsis AtHKT1*;1 Genotypes and Their Parental Strains. Mol. Plant.

[16] Liang W.J., Ma X.L., Wan P., Liu L.Y. (2018). Plant salt-tolerance mechanism: A review. Biochem. Biophys. Res. Commun..

[17] Stadtman E.R. (2006). Protein oxidation and aging. Free Radic. Res..

[18] Zong N., Li X.J., Wang L., Wang Y., Wen H.T., Li L., Zhang X., Fan Y.L., Zhao J. (2018). Maize *ABP2* enhances tolerance to drought and salt stress in transgenic *Arabidopsis*. J. Integr. Agric..

[19] Chen Y.E., Mao J.J., Sun L.Q., Huang B., Ding C.B., Gu Y., Liao J.Q., Hu C., Zhang Z.W., Yuan S. (2018). Exogenous melatonin enhances salt stress tolerance in maize seedlings by improving antioxidant and photosynthetic capacity. Physiol. Plant..

[20] Yu Z.P., Duan X.B., Luo L., Dai S.J., Ding Z.J., Xia G.M. (2020). How Plant Hormones Mediate Salt Stress Responses. Trends Plant Sci..

[21] Verma V., Ravindran P., Kumar P.P. (2016). Plant hormone-mediated regulation of stress responses. BMC Plant Biol..

[22] Chen K., Li G.J., Bressan R.A., Song C.P., Zhu J.K., Zhao Y. (2020). Abscisic acid dynamics, signaling, and functions in plants. J. Integr. Plant Biol..

[23] Niu M.L., Xie J.J., Chen C., Cao H.S., Sun J.Y., Kong Q.S., Shabala S., Shabala L., Huang Y., Bie Z.L. (2018). An early ABA-induced stomatal closure, Na^+^ sequestration in leaf vein and K^+^ retention in mesophyll confer salt tissue tolerance in *Cucurbita* species. J. Exp. Bot..

[24] Farhangi-Abriz S., Ghassemi-Golezani K. (2018). How can salicylic acid and jasmonic acid mitigate salt toxicity in soybean plants?. Ecotoxicol. Environ. Saf..

[25] Hazman M., Hause B., Eiche E., Nick P., Riemann M. (2015). Increased tolerance to salt stress in OPDA-deficient rice ALLENE OXIDE CYCLASE mutants is linked to an increased ROS-scavenging activity. J. Exp. Bot..

[26] Ahmad R.M., Cheng C., Sheng J., Wang W., Ren H., Aslam M., Yan Y.X. (2019). Interruption of Jasmonic Acid Biosynthesis Causes Differential Responses in the Roots and Shoots of Maize Seedlings against Salt Stress. Int. J. Mol. Sci..

[27] Wang J., Qin H., Zhou S.R., Wei P.C., Zhang H.W., Zhou Y., Miao Y.C., Huang R.F. (2020). The Ubiquitin-Binding Protein OsDSK2a Mediates Seedling Growth and Salt Responses by Regulating Gibberellin Metabolism in Rice. Plant Cell.

[28] Zhou J.H., Li Z.Y., Xiao G.Q., Zhai M.J., Pan X.W., Huang R.F., Zhang H.W. (2020). *CYP71D8L* is a key regulator involved in growth and stress responses by mediating gibberellin homeostasis in rice. J. Exp. Bot..

[29] Hyoung S., Cho S.H., Chung J.H., So W.M., Cui M.H., Shin J.S. (2020). Cytokinin oxidase *PpCKX1* plays regulatory roles in development and enhances dehydration and salt tolerance in *Physcomitrella patens*. Plant Cell Rep..

[30] Iglesias M.J., Terrile M.C., Bartoli C.G., D’Ippolito S., Casalongue C.A. (2010). Auxin signaling participates in the adaptative response against oxidative stress and salinity by interacting with redox metabolism in *Arabidopsis*. Plant Mol. Biol..

[31] Li Z.Y., Xu Z.S., He G.Y., Yang G.X., Chen M., Li L.C., Ma Y.Z. (2012). A mutation in *Arabidopsis* BSK5 encoding a brassinosteroid-signaling kinase protein affects responses to salinity and abscisic acid. Biochem. Biophys. Res. Commun..

[32] Fu J.J., Cheng Y.B., Linghu J.J., Yang X.H., Kang L., Zhang Z.X., Zhang J., He C., Du X.M., Peng Z.Y. (2013). RNA sequencing reveals the complex regulatory network in the maize kernel. Nat. Commun..

[33] Fernandez-Garcia N., Hernandez M., Casado-Vela J., Bru R., Elortza F., Hedden P., Olmos E. (2011). Changes to the proteome and targeted metabolites of xylem sap in *Brassica oleracea* in response to salt stress. Plant Cell Environ..

[34] Kakumanu A., Ambavaram M.M.R., Klumas C., Krishnan A., Batlang U., Myers E., Grene R., Pereira A. (2012). Effects of Drought on Gene Expression in Maize Reproductive and Leaf Meristem Tissue Revealed by RNA-Seq. Plant Physiol..

[35] Yu F., Liang K., Fang T., Zhao H.L., Han X.S., Cai M.J., Qiu F.Z. (2019). A group VII ethylene response factor gene, *ZmEREB180*, coordinates waterlogging tolerance in maize seedlings. Plant Biotechnol. J..

[36] Li C.H., Sun B.C., Li Y.X., Liu C., Wu X., Zhang D.F., Shi Y.S., Song Y.C., Buckler E.S., Zhang Z.W. (2016). Numerous genetic loci identified for drought tolerance in the maize nested association mapping populations. BMC Genom..

[37] Wang C.T., Yang Q.A., Yang Y.M. (2011). Characterization of the *ZmDBP4* gene encoding a CRT/DRE-binding protein responsive to drought and cold stress in maize. Acta Physiol. Plant..

[38] Bo C., Chen H.W., Luo G.W., Li W., Zhang X.G., Ma Q., Cheng B.J., Cai R.H. (2020). Maize WRKY114 gene negatively regulates salt-stress tolerance in transgenic rice. Plant Cell Rep..

[39] Delgado C., Mora-Poblete F., Ahmar S., Chen J.T., Figueroa C.R. (2021). Jasmonates and Plant Salt Stress: Molecular Players, Physiological Effects, and Improving Tolerance by Using Genome-Associated Tools. Int. J. Mol. Sci..

[40] Black C.C., Brown R.H., Moore R.C. (1978). Plant Photosynthesis.

[41] van Zelm E., Zhang Y.X., Testerink C. (2020). Salt Tolerance Mechanisms of Plants. Annu. Rev. Plant Biol..

[42] Wu T.Y., Wu X.Q., Xu X.Q., Kong W.L., Wu F. (2020). Salt Tolerance Mechanism and Species Identification of the Plant Rhizosphere Bacterium JYZ-SD2. Curr. Microbiol..

[43] Abbasi H., Jamil M., Haq A., Ali S., Ahmad R., Malik Z., Parveen Z. (2016). Salt stress manifestation on plants, mechanism of salt tolerance and potassium role in alleviating it: A review. Zemdirbyste.

[44] Wang Y., Huang L., Du F., Wang J., Zhao X., Li Z., Wang W., Xu J., Fu B. (2021). Comparative transcriptome and metabolome profiling reveal molecular mechanisms underlying *OsDRAP1*-mediated salt tolerance in rice. Sci. Rep..

[45] Duarte-Delgado D., Said D., Schoof H., Oyiga B.C., Schneider M., Mathew B., Léon J., Ballvora A. (2020). Transcriptome profiling at osmotic and ionic phases of salt stress response in bread wheat uncovers trait-specific candidate genes. BMC Plant Biol..

[46] Umezawa T., Sugiyama N., Mizoguchi M., Hayashi S., Myouga F., Yamaguchi-Shinozaki K., Ishihama Y., Hirayama T., Shinozaki K. (2009). Type 2C protein phosphatases directly regulate abscisic acid-activated protein kinases in *Arabidopsis*. Proc. Natl. Acad. Sci. USA.

[47] Thalmann M., Pazmino D., Seung D., Horrer D., Nigro A., Meier T., Kolling K., Pfeifhofer H.W., Zeeman S.C., Santelia D. (2016). Regulation of Leaf Starch Degradation by Abscisic Acid Is Important for Osmotic Stress Tolerance in Plants. Plant Cell.

[48] Ye H.Y., Du H., Tang N., Li X.H., Xiong L.Z. (2009). Identification and expression profiling analysis of TIFY family genes involved in stress and phytohormone responses in rice. Plant Mol. Biol..

[49] Valenzuela C.E., Acevedo-Acevedo O., Miranda G.S., Vergara-Barros P., Holuigue L., Figueroa C.R., Figueroa P.M. (2016). Salt stress response triggers activation of the jasmonate signaling pathway leading to inhibition of cell elongation in *Arabidopsis* primary root. J. Exp. Bot..

[50] Iglesias M.J., Terrile M.C., Windels D., Lombardo M.C., Bartoli C.G., Vazquez F., Estelle M., Casalongue C.A. (2014). MiR393 regulation of auxin signaling and redox-related components during acclimation to salinity in *Arabidopsis*. PLoS ONE.

[51] Wang Y., Shen W., Chan Z., Wu Y. (2015). Endogenous Cytokinin Overproduction Modulates ROS Homeostasis and Decreases Salt Stress Resistance in *Arabidopsis Thaliana*. Front. Plant Sci..

[52] Jiang K., Moe-Lange J., Hennet L., Feldman L.J. (2016). Salt Stress Affects the Redox Status of *Arabidopsis* Root Meristems. Front. Plant Sci..

[53] Cai R.H., Dai W., Zhang C.S., Wang Y., Wu M., Zhao Y., Ma Q., Xiang Y., Cheng B.J. (2017). The maize WRKY transcription factor *ZmWRKY17* negatively regulates salt stress tolerance in transgenic *Arabidopsis* plants. Planta.

[54] Sun J.Q., Jiang H.L., Xu Y.X., Li H.M., Wu X.Y., Xie Q., Li C.Y. (2007). The CCCH-type zinc finger proteins *AtSZF1* and AtSZF2 regulate salt stress responses in *Arabidopsis*. Plant Cell Physiol..

[55] Lee S.K., Kim B.G., Kwon T.R., Jeong M.J., Park S.R., Lee J.W., Byun M.O., Kwon H.B., Matthews B.F., Hong C.B. (2011). Overexpression of the mitogen-activated protein kinase gene *OsMAPK33* enhances sensitivity to salt stress in rice (*Oryza sativa* L.). J. Biosci..

[56] Mehlmer N., Wurzinger B., Stael S., Hofmann-Rodrigues D., Csaszar E., Pfister B., Bayer R., Teige M. (2010). The Ca^2+^-dependent protein kinase CPK3 is required for MAPK-independent salt-stress acclimation in *Arabidopsis*. Plant J..

[57] Liu L., Chen J., Tan Y.N., Zhou T.S., Ouyang N., Zeng J., Yuan D.Y., Duan M.J. (2019). Increasing Fatty Acids in Rice Root Improves Silence of Rice Seedling to Salt Stress. Rice Sci..

[58] Zhang X.G., Cai H.L., Lu M., Wei Q.Y., Xu L.J., Bo C., Ma Q., Zhao Y., Cheng B.J. (2019). A maize stress-responsive Di19 transcription factor, *ZmDi19*-1, confers enhanced tolerance to salt in transgenic *Arabidopsis*. Plant Cell Rep..

[59] Kim D., Landmead B., Salzberg S.L. (2015). HISAT: A fast spliced aligner with low memory requirements. Nat. Methods.

[60] Anders S., Pyl P.T., Huber W. (2015). HTSeq-a Python framework to work with high-throughput sequencing data. Bioinformatics.

[61] Marioni J.C., Mason C.E., Mane S.M., Stephens M., Gilad Y. (2008). RNA-seq: An assessment of technical reproducibility and comparison with gene expression arrays. Genome Res..

[62] Love M.I., Huber W., Anders S. (2014). Moderated estimation of fold change and dispersion for RNA-seq data with DESeq2. Genome Biol..

[63] Zhao Y., Hu F.X., Zhang X.G., Wei Q.Y., Dong J.L., Bo C., Cheng B.J., Ma Q. (2019). Comparative transcriptome analysis reveals important roles of nonadditive genes in maize hybrid An’nong 591 under heat stress. BMC Plant Biol..

[64] Livak K.J., Schmittgen T.D. (2001). Analysis of relative gene expression data using real-time quantitative PCR and the 2(T)(-Delta Delta C) method. Methods.

