# Supplementary material for: Comparative Transcriptomics Reveals the Molecular Mechanism of the Parental Lines of Maize Hybrid An’nong876 in Response to Salt Stress
Source: Int J Mol Sci. 2022 May 7;23(9):5231. doi: 10.3390/ijms23095231 (PMC9100555; doi:10.3390/ijms23095231)
Supplement: Supplementary file 1 [file ijms-23-05231-s001.zip › ijms-1677058-supplementary.pdf]

Supplementary Materials:

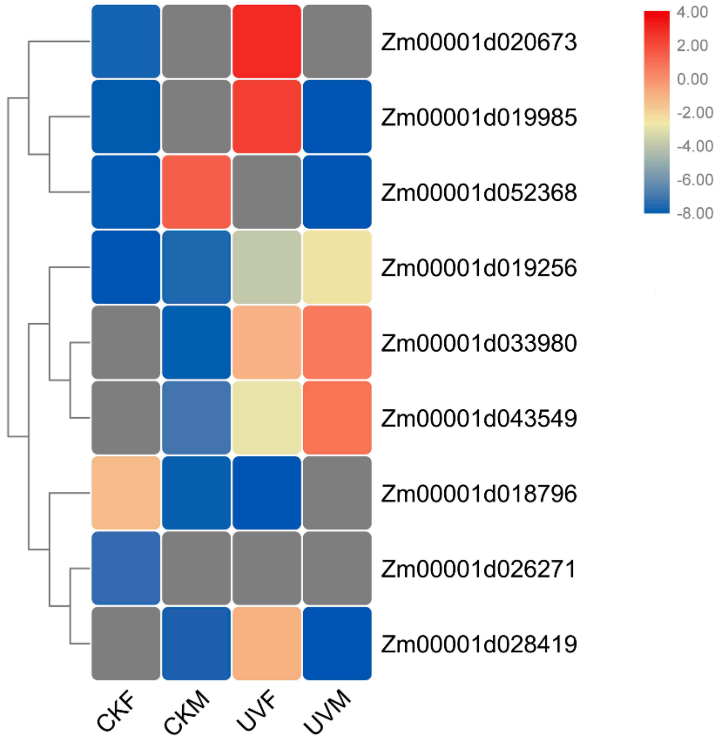

Figure S1. Heatmap of relative expression level of 9 selected DEGs by quantitative RT-PCR validation. The colored bars represent the gene values of Log_2(qRT-PCR).

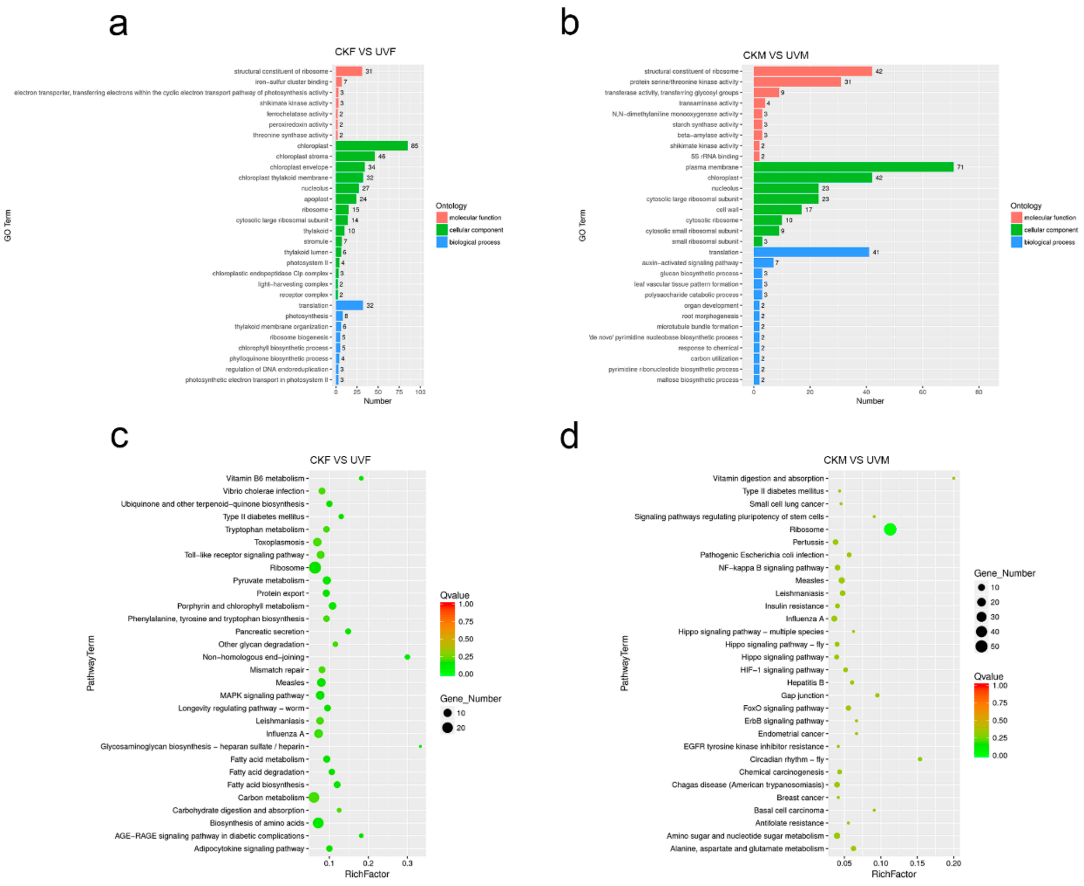

Figure S2. GO enrichment and KEGG enrichment pathway of the Genotype-specific DEGs of CKF vs.

UVF and CKM vs. UVM comparisons. (**a**) GO enrichment of CKF vs. UVF comparison. (**b**) GO enrichment of CKM vs. UVM comparison. (**c**) KEGG enrichment of CKF vs. UVF comparison. (**d**) KEGG enrichment of CKM vs. UVM comparison.

Table S1. Statistics of RNA-seq results.

Samples	Raw reads	Clean reads	Q20 (%)	Q30 (%)	GC (%)	Total mapped (%)
CKF1	45234388	44981596	95.25	86.63	56.36	78.47
CKF2	44879052	44608876	95.01	86.06	56.13	77.94
CKF3	45858280	45588504	94.84	85.71	56.05	77.45
CKM1	44141640	43877310	94.7	85.47	57.31	76.19
CKM2	45840656	45532112	94.49	84.99	57.59	76.22
CKM3	44633574	44352042	94.85	85.69	56.6	76.62
UVF1	44103196	43854056	95.64	87.49	55.2	78.26
UVF2	44929308	44679960	95.32	86.71	55.32	78.94
UVF3	44073946	43819472	95.21	86.54	55.66	77.88
UVM1	45504310	45201426	94.9	85.83	56.21	75.88
UVM2	45747746	45460920	95.22	86.62	56.42	77.25
UVM3	44211918	43919168	94.6	85.2	56.56	75.8

Table S2. Common DEGs of CKF vs. UVF and CKM vs UVM comparisons involved in photosynthesis.

Gene ID	$Log_2FoldChange$ (CKF vs UVF)	$Log_2FoldChange$ (CKM vs UVM)
Zm00001d006540	-4.1528	-3.362937
Zm00001d003767	-4.103649	-1.752805
Zm00001d034543	-2.67494	-1.727834
Zm00001d005996	-3.29273	-1.941736
Zm00001d041819	-2.775837	-1.630673
Zm00001d005446	-2.329359	-1.718646
Zm00001d018779	-3.885081	-1.653844
Zm00001d013146	-2.936356	-1.647571
Zm00001d021703	-2.690981	-1.582301
Zm00001d038984	-2.800758	-1.313408
Zm00001d013039	-3.260297	-1.615644
Zm00001d034283	-2.052624	-1.380715
Zm00001d008706	-3.032207	-2.156678
Zm00001d036535	-1.960853	-1.395406
Zm00001d027422	-2.875056	-1.541906
Zm00001d020877	-2.746022	-1.507007
Zm00001d035003	-2.576361	-2.01067
Zm00001d043299	-2.548225	-1.775833
Zm00001d016943	-2.957938	-2.021857

Zm00001d018069	-1.982485	-1.770603
Zm00001d052242	-1.461311	-1.257588
Zm00001d011833	-1.783568	-1.331641
Zm00001d050889	-1.318194	-1.538137
Zm00001d023713	-1.79474	-2.040135
Zm00001d035859	-1.664865	-1.715635
Zm00001d000272	-1.328206	-1.239919

Table S3. Genotype-specific DEGs participated in photosynthesis (CKF vs UVF).

Gene ID	log_2FoldChange
Zm00001d014564	-1.344234
Zm00001d035135	-1.998215
Zm00001d042049	-1.934934
Zm00001d012293	5.3314446
Zm00001d013534	-1.295529
Zm00001d034760	1.0353944
Zm00001d000417	-1.452796
GRMZM5G856777	-1.693664

Table S4. Genotype-specific DEGs participated in photosynthesis (CKM vs UVM).

Gene ID	log_2FoldChange
Zm00001d018797	-2.475191
Zm00001d047789	-1.730859
Zm00001d035002	-1.568683
Zm00001d019518	-1.823367
Zm00001d021620	-1.356876
Zm00001d010715	-1.083137
Zm00001d042175	-1.419744

Table S5. Primers used for qRT-PCR in this study.

Primer name	Primer sequence
Zm00001d018796-F	CACAATAAACCCAGCAAGAG
Zm00001d018796-R	AACCCCTGGAGGAAGTTT
Zm00001d019256-F	GTGTGGAGGATTGGAGTTTT
Zm00001d019256-R	CGCACTTTCTCCTTCAAATC
Zm00001d019985-F	CAGTACTTCAAGTCCACCAAGGCA
Zm00001d019985-R	GGCGAAGACGAATAGGTCGTTGCT
Zm00001d020673-F	ACTTCTTCGATCCGATCATC
Zm00001d020673-R	AAGACTTGACAGTTTGCAGG
Zm00001d026271-F	GTGCCAACAAGCATGCTGCG
Zm00001d026271-R	CATGTTGAGGTCGAACCCCA
Zm00001d028419-F	ACCCAAGAACGACTTTCTG
Zm00001d028419-R	TCCCAGCAGTGAATAAATCC
Zm00001d033980-F	CTTTGAGGTCCTGGACAAAGACC

Zm00001d033980-R	TGATCATCTGTTCAACCTCCTCG
Zm00001d043549-F	CAAGGAGTACAACCTCACCAG
Zm00001d043549-R	GATGGAGCAGTTCATCTTGATG
Zm00001d052368-F	GATCTCCAAGATGACGGATC
Zm00001d052368-R	TTGGCCTTAGACTTCATGAC
GAPDH-F	ATCAACGGCTTCGGAAGGAT
GAPDH-R	CCGTGGACGGTGTCGTACTT
